# Epiphytic and Endophytic Fungi Colonizing Seeds of Two *Poaceae* Weed Species and *Fusarium* spp. Seed Degradation Potential In Vitro

**DOI:** 10.3390/microorganisms11010184

**Published:** 2023-01-11

**Authors:** Jevgenija Ņečajeva, Anete Borodušķe, Vizma Nikolajeva, Māris Seņkovs, Ineta Kalniņa, Ance Roga, Edmunds Skinderskis, Dāvids Fridmanis

**Affiliations:** 1 Institute of Plant Protection Research ‘Agrihorts’, Latvia University of Life Sciences and Technologies, Paula Lejiņa Iela 2, LV-3004 Jelgava, Latvia; 2 Microbial Strain Collection of Latvia, Faculty of Biology, University of Latvia, Jelgavas Iela 1, LV-1004 Rīga, Latvia; 3 Latvian Biomedical Research and Study Centre, Rātsupītes Iela 1, LV-1067 Rīga, Latvia

**Keywords:** *Echinochloa crus-galli*, barnyard grass, *Avena fatua*, wild oat, *Fusarium*, seed-borne fungi, soil seed bank, arable weeds, biocontrol

## Abstract

Fungi colonizing the surface and endosphere of two widespread *Poaceae* weed species, *Avena fatua* and *Echinochloa crus-galli*, were isolated to compare the taxonomic composition between the plant species, location, and year of the seed collection. The seed-degrading potential of *Fusarium* isolated from the seeds was tested by inoculating seeds of *E. crus-galli* with spore suspension. Molecular identification of epiphytic and endophytic fungal genera was performed by sequencing the ITS region of rDNA. Endophytes comprised of significantly lower fungal richness compared to epiphytes. A significant taxonomic overlap was observed between the endosphere and seed surface. The most abundant genera were *Alternaria*, *Fusarium*, *Cladosporium,* and *Sarocladium*. Analysis of similarities and hierarchical clustering showed that microbial communities were more dissimilar between the two plant species than between the years. *Fusarium* isolates with a high potential to infect and degrade *E. crus-galli* seeds in laboratory conditions belong to *F. sporotrichioides* and *F. culmorum*.

## 1. Introduction

Arable weeds are one of the major sources of crop yield loss, reaching as high as 32% of actual yield loss and potentially amounting to 71% if no weed control is implemented [1]. Management of the inactive weed propagules that include soil seed banks for all the annual weed species is one of the tasks of integrated weed management strategies [2]. Current agricultural policy in the EU formulated in the ‘Farm to Fork strategy’ requires reducing the use of pesticides. Therefore, increasing attention is being paid to ecological interactions between crops, weeds, and other organisms in the agroecosystems that can facilitate pest control for sustainable crop production. Natural seed degradation aided by soil microbial communities offers a means for weed biocontrol. Seed microbiota are largely derived from soil microbial communities [3], yet partial vertical transmission from parental plants has also been reported [4], and microbiota can contain beneficial microorganisms that promote seed longevity and seedling survival [5], as well as pathogenic microorganisms leading to seed degradation [6].

Seeds that are quiescent or dormant (not germinating) have physical and chemical means of defense against pathogenic microorganisms [7,8]. A hard seed coat that constitutes the mechanical defense is correlated with a lower decay rate of seeds [6]. Phenolic compounds in the seed, seed coat, or specifically in the seed hull in a broad variety of species are an important part of seed chemical defenses [9]. The presence of these compounds may vary depending on the origin of the seeds as well as growth and seed maturation conditions. Moreover, if regarded as a species-specific feature, an association with certain bacteria that are antagonistic to pathogenic fungi or fungi that are antagonistic to other pathogens can be considered a defense mechanism [10]. Weed seeds differ in their ability to withstand microbial attack. Both inter- and intraspecific differences include seed-coat thickness [11], the chemical composition of the seed coat and other seed structures, and, probably, enzymatic defense mechanisms. The antifungal activity of a specific peptide isolated from *E. crus-galli* seeds was tested on several plant pathogens and had a pronounced effect on *Fusarium graminearum, F. oxysporum, F. solani,* and *F. verticillioides* (current name *F. fujikuroi*) [12], suggesting that seeds of *E. crus-galli* have limited susceptibility to these fungal pathogens. However, other *Fusarium* species were not tested. The presence of pathogen antagonists on the seed surface or in the endosphere is another potentially important factor for seed defense. It is, therefore, important to compare the resistance to microbial decay in seeds from either different parent plants or different populations.

The action of potentially seed-degrading microorganisms can be species-specific. For example, seeds of *Pseudotsuga menziesii* were shown to carry numerous *Fusarium* species that mostly caused no disease of either seeds or plants [13]. At the same time, *F. oxysporum* significantly reduced the germination of *Pinus sylvestris* and *P. pinea*, while *F. verticillioides* (*F. fujikuroi*) only reduced the germination of *P. sylvestris* [14]. The genus *Fusarium* includes species and strains that are pathogenic to seeds of different species of *Poaceae*. Soil-borne *Fusarium* species can cause significant seed mortality in *Bromus tectorum* [15] as well as *Avena fatua* [16]. The susceptibility of the plants or seeds to fungal attack can depend on the particular strain of the fungal species. This was demonstrated for soil-borne *F. oxysporum* isolates infecting *E. crus-galli* [17] and seed-borne *F. oxysporum* isolates with different potentials to infect *Diplotaxis* or *Eruca* plants [18]. Seed-borne fungi from the *F. tricinctum* species complex have also been shown to produce toxic metabolites that inhibit seedling growth in *Bromus tectorum* [19].

In addition to the soil-borne microorganisms, seeds and seedlings can be affected by the seed-borne pathogens from mature seeds. Seed-borne pathogens are a major source of disease in cultivated plants, including grains, and seeds can be vectors that transport pathogenic microorganisms between different locations [20].

Wild oat (*Avena fatua* L.) is a highly economically detrimental weed species distributed in cereal-growing areas worldwide. All parts of *A. fatua* seeds—awns, lemma, palea, and caryopsis—were reported to harbor yeasts and filamentous fungi belonging to 10 different genera [21]. The most frequent genera were *Alternaria*, *Cladosporium,* and *Drechslera*, while *Fusarium* was comparatively frequent on lemma and caryopses. Barnyard grass (*Echinochloa crus-galli* (L.) Beauv.) is also a widespread weed species that causes significant yield loss in different crops. To the best of our knowledge, the seed-borne fungi of *E. crus-galli* seeds have not been characterized, but in a closely related species, *E. glabrescens*, endophytes were described in different tissues, including seeds, of plants collected in different locations [22]. The rate of colonization of seeds was the second highest among different tissue types, after lower leaf blade.

Sustainable control of barnyard grass populations in arable lands is an important goal. Numerous cases of herbicide resistance have been reported for different varieties of this species [23]. An alternative to increased herbicide use is the reduction and management of the soil seed bank [24,25]. In this study, we chose *E. crus-galli* as a model organism to test the ability of fungi to degrade seeds under laboratory conditions because these plants produce large amounts of seeds, providing abundant material for experiments. Seeds are dormant at maturation, although dormancy can be partly released during dry storage [26].

This study aimed to compare the diversity and taxonomic composition of seed-borne fungi from mature seeds of *A. fatua* and *E. crus-galli* collected in different populations and to test the ability of epiphytic and endophytic seed-borne *Fusarium* spp. to infect and degrade seeds of *E. crus-galli* in laboratory conditions.

## 2. Materials and Methods

### 2.1. Study Sites and Seed Collection

Seeds were collected at maturity in three locations in Latvia in 2020 and the same locations in 2021. In 2020, *A. fatua* seeds were collected in one additional location (Table 1). Seeds from 750 *A. fatua* plants and 350 *E. crus-galli* plants were collected by shaking the seeds into sterile containers and storing them at 4–5 °C. Fungi were isolated within 24 h from seed collection. Additional samples of *E. crus-galli* seeds were dried over silica gel at ambient temperature for 14–21 days to reach moisture content below 15% and stored at −20 °C until further use in the inoculation experiment.

### 2.2. Isolation of Fungi

The isolation of seed epiphytes was carried out by agitating the seeds in sterile 10 mM MgSO_4_ (50 mL) and isolating epiphytic fungi from the acquired rinsing solution on 10% yeast malt agar medium (YMA; full medium: glucose, 10 g L^−1^; yeast extract, 3 g L^−1^; peptone, 5 g L^−1^; malt extract, 3 g L^−1^; all chemicals were produced by BioRad (Le Pond de Claix, France), except glucose (Duchefa, Haarlem, The Netherlands). Agar: 15 g L^−1^, Scharlau, Spain) supplemented with chloramphenicol (0.1 g L^−1^, Sigma-Aldrich, Shanghai, China) to prevent bacterial growth using dilution to extinction method (dilution range from 10^−2^ to 10^−4^) [27]. Afterward, the seeds were surface-sterilized by immersing them in 70% ethanol for 1 min, rinsing once with sterile deionized water, then immersing them in 10% commercial bleach (ACE, c.a. 5% Na hypochlorite) for 10 min, rinsing twice with sterile deionized water and rinsing with 70% ethanol and three times with sterile deionized water. Endophytic fungi were isolated from surface-sterilized seeds cut in halves and incubated on 10% YMA medium. A total of 10 *A. fatua* and 20 *E. crus-galli* seeds per population per collection year were used. Pure cultures were obtained from the colonies that developed on the 10% YMA medium, and mycelia were cultivated on 100% YMA medium until identification. One sample of each morphotype was used for Sanger-sequencing-based taxonomic identification.

### 2.3. Identification of Fungi

As one of the most universal approaches, ITS region sequencing was used as our primary means for taxonomic identification to at least the genus level. For that purpose, we performed DNA extraction by homogenizing ~10 µg of fungal material in lysis buffer (containing 2.5% PVP) with Lysing Matrix D (MP Biomedicals, Eschwege, Germany). The bead beating was carried out for 2 × 30s in FastPrep^®^-24 Classic bead beating grinder and lysis system instrument (MP Biomedicals, Eschwege, Germany). Sample-containing tubes were then incubated at 56 °C for 30 min and centrifuged at 5600 g for 20 min to precipitate cell debris. Further, microbial DNA was isolated from 400 µL of lysate with NucleoMag^®^ 96 Plant kit (Macherey-Nagel) following the manufacturer’s instructions. The amplification of ITS region by PCR was carried out in total volume of 12 µL and using Phire Plant Direct PCR Master Mix (Thermo Fisher Scientific, Waltham, MA, USA), which was supplemented with 5 pmol of ITS5—Fw (5′-GGAAGTAAAAGTCGTAACAAGG-3′) and ITS4—Rs (5′-TCCTCCGCTTATTGATATGC-3′) primers [28] and 1 µL of fungal DNA. The created PCR mixture was then incubated on a VeritiTM 96-well thermal cycler (Applied Biosystems, Waltham, MA, USA) in following temperature regime: 5 min of initial denaturation at 98 °C, 40 product amplification cycles of 5 s at 98 °C, 5 s at 55 °C, and 20 s at 72 °C, and whole procedure was finalized by 1 min extension step at 72 °C. The excess of dNTPs and primers was then removed by treatment with Exonuclease I (0.5 µL (Thermo Fisher Scientific) and Shrimp Alkaline Phosphatase (2 µL) (Thermo Fisher Scientific) for 40 min at 37 °C, and the enzymes were inactivated through incubation at 95 °C for 20 min. Afterward, 1 µL of the purified samples was transferred to BigDye^®^ Terminator v3.1 Cycle Sequencing reaction mixture (Applied Biosystems), which was supplemented with one of the previously used primers. Sequencing products were analyzed on 3130xl Genetic Analyzer (Applied Biosystems), which acquired sequences manually assembled and curated using SeqMan Pro from Lasergene 14 software package (DNASTAR Inc., Madison, WI, USA). BLAST analysis of acquired sequences against NCBI nucleotide database was then used for taxonomic identification of fungal isolate. Additionally, we used Protax-Fungi web-based tool [29] to search for matching ITS sequences in the Unite database.

Frequency of occurrence was calculated as the summary incidence in seed samples from each species population in each year divided by the total number of seed samples, seven in *A. fatua* and six in **E. crus-galli*.*

Since, in the case of *Fusarium*, ITS analysis does not provide sufficient taxonomic resolution to perform identification at the species level, additional TEF-1α region sequencing analysis was performed for isolates classified as members of this genus. All procedures, in this case, were essentially identical to those of ITS analysis. The only exception was the employment of EF1T (5′-ATGGGTAAGGAGGACAAGAC-3′) and EF2T (5′-GGAAGT ACCAGTGATCATGTT-3′) primers [30] in both PCR and sequencing reactions. All described molecular-biology-related activities were carried out at ‘Genome center’–a genetic analysis core facility of Latvian Biomedical Research and Study center.

### 2.4. Artificial Inoculation of Seeds

Isolates identified as *Fusarium* spp. were cultivated on Potato Dextrose Agar medium (Biolife) at room temperature (22 ± 3 °C) until sporulation was observed. Five 90 mm Petri dishes were used for each isolate. When sporulation was attained, spores were harvested by pouring 10 mL of sterile water into each Petri dish, gently scraping the mycelium with a sterile microbiological loop, and collecting suspension from all five dishes in a 50 mL centrifuge tube. The suspension was centrifuged for 5 min at 500 g, and the supernatant was removed, leaving 5 mL of the suspension. Spores were counted using a hemocytometer to calculate the spore concentration. Subsequently, the suspension was diluted to the concentration of 250,000 spores mL^−1^. If the concentration of spores was too low, cultivation of the isolate was repeated.

To assess the potential of seed-borne *Fusarium* isolates to infect and degrade seeds from different populations, surface-sterilized seeds of each of the three populations collected in 2020 and 2021 (procedure described above, with the only difference being the usage of undiluted bleach) were inoculated with a fungal spore suspension by immersing the seeds in the spore suspension in sterile plastic tubes and vortexing for one min. After that, seeds were placed on 0.7% water agar gel (Duchefa) in sterile 60 mm Petri dishes and sealed with Parafilm. There were three Petri dishes per isolate, each containing 20 seeds. Non-inoculated seeds placed on water agar gel were used as a control. The inoculated seeds were incubated in the dark at 10 °C for 14 days to prevent rapid seed germination and then incubated for additional 28 days at room temperature (25 ± 3 °C). Seeds were scored as infected if *Fusarium* hyphae were observed on the seed surface. At the end of the incubation period, a cut test was performed to identify decayed seeds. Seeds were considered dead if the seed embryo was discolored or decayed. Seedlings were scored as infected if tufts of white, pink, or red hyphae were observed on the coleoptile or brown spots and stunted growth of the coleoptile were observed [31]. Otherwise, seedlings were scored as non-infected.

### 2.5. Statistical Analysis

Statistical analysis of the data was performed in R 4.1.1. Data from different populations of each species were used to create a presence/absence table for different fungal genera isolated from the seeds. Jaccard distance matrix was created using ‘vegdist()’ function from the ‘vegan’ package [32]. Taxonomic composition was compared between seeds of each of the two species collected in each year by hierarchical clustering based on Jaccard distances and creating a heatmap using function ‘pheatmap()’ in the ‘pheatmap’ package [33]. To compare the effect of species and seed-collection year, analysis of similarities was performed using the function ‘anosim()’ from the ‘vegan’ package using the same presence/absence matrix. Binomial logistic regression was used to analyze the ability of *Fusarium* isolates to infect *E. crus-galli* seeds: the effect of seed-collection year, population, and the fungal isolate on the number of intact seeds in the samples were tested. Multiple comparisons were performed using the ‘glht()’ function in the ‘multcomp’ package [34]. Venn diagrams were prepared using the ‘VennDiagram’ package [35].

## 3. Results

### 3.1. The Diversity of Epiphytic Seed-Borne Fungi Is Greater Than That of the Endophytic Seed-Borne Fungi

**Figure 1:**
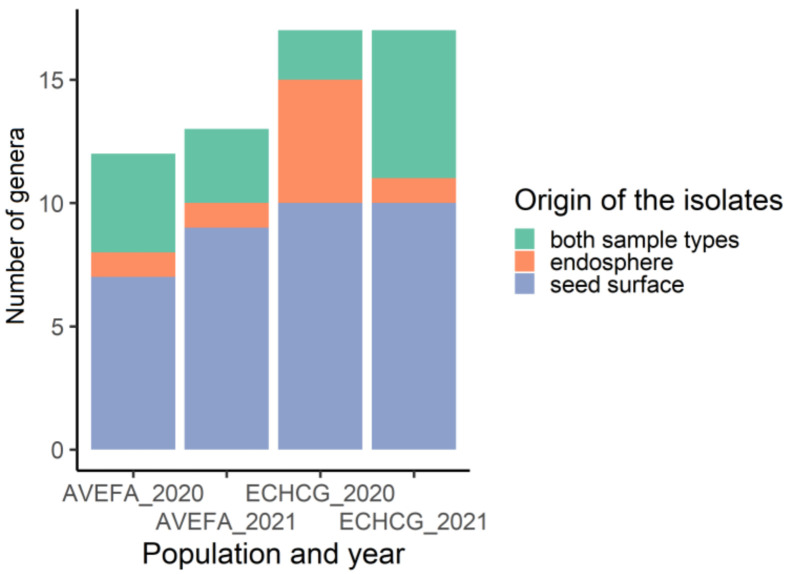
Number of fungal genera isolated from the seed surface, seed endosphere, as well as from both endosphere and seed surface of *A. fatua* (AVEFA) and *E. crus-galli* (ECHCG) in 2020 and 2021.

In total, 72 morphologically unique fungal isolates were recovered in 2020, and 96 were recovered in 2020 and 2021 from seeds of both *A. fatua* and *E. crus-galli*. Overall richness was higher in samples isolated from *E. crus-galli* (Figure 1).

The most frequent genera, found in more than one population of each species, were *Alternaria, Fusarium, Cladosporium,* and *Sarocladium*, followed by *Sporobolomyces, Epicoccum, Vishniacozyma,* and *Aureobasidium*. Several isolates were identified at the species level. The list of genera and species (identified at the species level) is given in Table 2.

**Figure 2:**
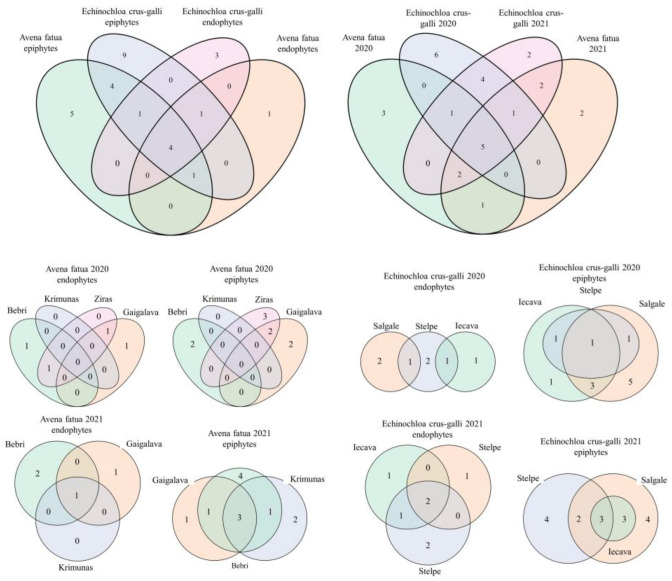
Venn diagrams showing the numbers of genera overlapping between different seed samples and habitats (epiphytes/endophytes).

*Alternaria*, *Cladosporium*, *Fusarium*, and *Sarocladium* were present in the seed endosphere, as well as on the seed surface of both plant species. However, most fungal isolates were either uniquely present in one plant species or one of the years (Figure 2). Different collection sites yielded a similar number of fungal isolates, and a rough proportion of 1:2 in the number of endophytic to epiphytic isolates was maintained in both years.

Three to six genera were isolated exclusively from the seeds of one species collected in one of the years. The number of genera isolated from the endosphere only was three in the case of *E. crus-galli*, while in *A. fatua*, only one genus was isolated from the endosphere only. Five genera were isolated only from the *A. fatua* seed surface, while nine were isolated from the *E. crus-galli* seed surface, and four more genera were isolated from the surface of both species. Comparing the number of common genera in seeds collected from different locations, it was found that more were common in 2021 in both species and either isolated from the seed surface or the endosphere, while in 2020, no common genera were found in *A. fatua* from different locations, and only one was common to all three locations among the isolates from the *E. crus-galli* seed surface. The highest number of genera exclusive to one population was isolated from the *E. crus-galli* seed surface in 2020, where five were unique to seeds from the Salgale location (Figure 2). These results indicate that the seed mycobiota of the studied species differ between plant populations.

### 3.2. Taxonomic Composition of Seed-Borne Fungi Differs between the Two Species

**Figure 3:**
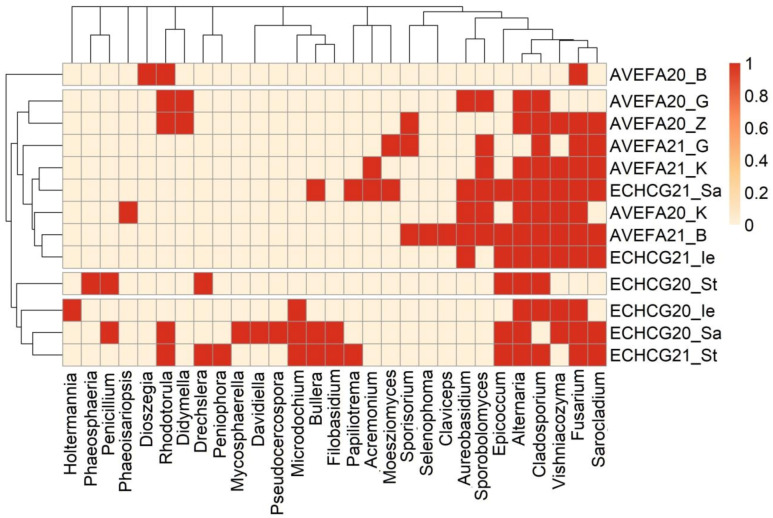
Hierarchical cluster diagram and heatmap based on Jaccard distances between fungal communities isolated from *A. fatua* (AVEFA) and *E. crus-galli* (ECHCG) seed samples collected in 2020 and 2021 in different locations (indicated by letters).

*E. crus-galli* mycobiota exhibited higher year-to-year variability compared to *A. fatua*. The hierarchical clustering based on Jaccard distances revealed the grouping of the *A. fatua* samples from both collection years, while *E. crus-galli* samples also clustered together, except for samples from seeds collected in 2021, revealing some overlap between the species (Figure 3).

The results of cluster analysis were supported by the analysis of similarities. Dissimilarity was not significant between the collection years, but there was a significant dissimilarity (*p* = 0.013) between the two species, although a great degree of overlap (R = 0.218) was also observed.

### 3.3. F. Sporotrichioides and F. Culmorum Had the Highest Potential to Reduce the Number of Intact E. Crus-Galli Seeds

Ten *Fusarium* isolates for which sporulation was attained were chosen for inoculation experiments using *E. crus-galli* seeds (Table 3).

**Figure 4:**
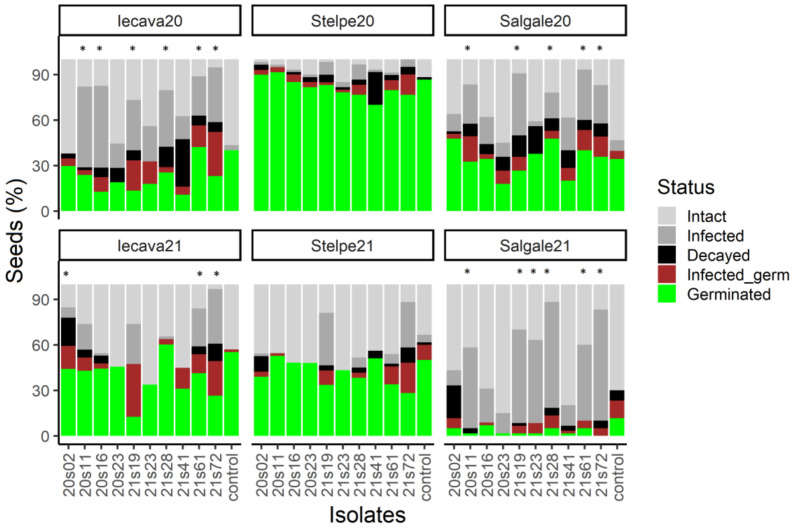
Seed fate after inoculating surface-sterilized seeds with spore suspension of different *Fusarium* isolates and subsequent incubation at 10 °C for 14 days and at room temperature for 28 days. Non-inoculated seeds were used as a control. Seeds collected in different years and from different plant populations are shown separately. Asterisk (*) denotes treatments where the number of intact seeds at the end of experiment was significantly different from the control treatment.

Although all collected seeds were initially dormant, dormancy was partially lost in the drying process, and germination was stimulated by surface sterilization. The number of germinated seeds varied from 1.7% to 94.8% between the combinations of the collection year and plant population and between seeds infected with different isolates. Similarly, the proportion of infected and decayed seeds, as well as seedlings infected after germination, was variable between the treatments: 0–73.3% (infected seeds), 0–31.1% (decayed seeds), and 0–34.9% (infected seedlings) (Figure 4).

Inoculation of seeds with spores of the isolates 20s11, 21s19, 21s28, 21s61, and 21s72 resulted in the lowest number of intact seeds (25% or less of all the seeds remained intact) in all combinations of the collection year and plant population (Figure 4). The number of infected and decayed seeds was inconsistent between the seed-collection years and populations, except for isolates 21s19 and 21s72. Although the number of intact seeds was not significantly different from the control in the Stelpe population, including in 2020 and 2021, there was a tendency towards a lower number of intact seeds in samples inoculated with 21s19 and 21s72 (in 2021, the difference between 21s72 and the control was close to significant, *p* = 0.0622). Infected seeds were present in the control treatment, indicating that either the sterilization method was insufficient to eliminate all fungi from the seed surface or that *Fusarium* fungi were present deeper in the seed coat tissues.

**Figure 5:**
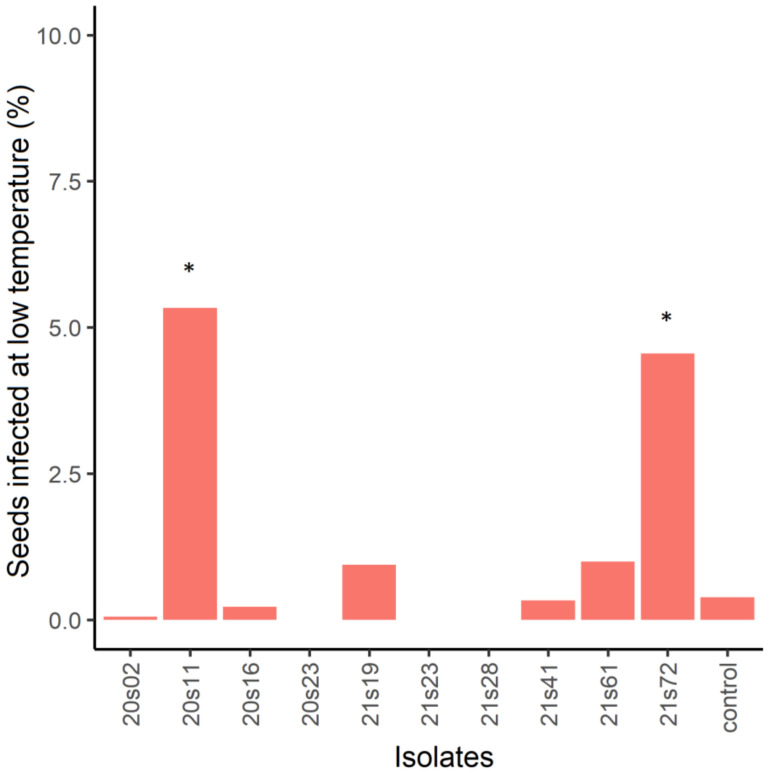
Average number of seeds with signs of *Fusarium* infection after 14 days at 10 °C. Non-inoculated seeds were used as a control. Asterisk (*) denotes treatments where the number of intact seeds at the end of experiment was significantly different from the control treatment.

Two 20s11 and 21s72 isolates identified as *F. sporotrichioides* could infect seeds already at 10 °C. On average, 5% of the total number of seeds were infected at low temperatures (Figure 5).

We observed competitive interaction between *Fusarium* isolates applied for seed inoculation and fungal isolates emerging from seeds in seed decay tests. Most antagonistic isolates were identified morphologically as *Alternaria*.

## 4. Discussion

### 4.1. Taxonomic Composition of Seed Fungal Communities

In this study, a culture-based taxonomic assessment of mycobiota of seeds from two noxious weed species, *A. fatua* and *E. crus-galli*, was performed. Fungal communities were compared with regard to plant species, seed-collection years, and locations, as well as the habitats (seed surface or seed endosphere). More seed-borne fungi were isolated from the surface of non-sterilized seeds of both weed species compared to the seed endosphere. Although the surface-sterilization method used in this study may not have eliminated 100% of the surface-borne microorganisms, there was a clear reduction in the number of isolated genera.

Most isolated fungal genera and species were typical for the mycobiota of seeds [5]. Two genera, *Fusarium* and *Alternaria*, were ubiquitously represented in endophytic and epiphytic samples from both weed species. *Alternaria* is a common seed colonizer [36,37]. Certain isolates of *Alternaria* have previously been reported to suppress the germination of *E. crus-galli* and *A. fatua* [38]. Thus, *Alternaria alternata* and *A. tenuissima* strains have been proposed as biocontrol agents for this weed species. In addition to its seed-degrading capacity, *Alternaria* sp. effectively infects *E. crus-galli* seedlings, precluding further development [39]. *Fusarium* is one of the most widespread seed-associated fungal genera [36] and is a genus represented by numerous species of common cereal pathogens. *Fusarium* is often isolated from *Poaceae* seeds [40]. Due to pathogenic properties, members of this genus are often viewed as a target rather than an agent of biocontrol. However, specific strains of *F. avenaceum* [41] and *F. culmorum* [16] have been shown to successfully colonize and degrade caryopsis of *A. fatua*, suggesting that certain strains of *Fusarium* are potentially interesting for application in weed seed biocontrol.

*Cladosporium* is another widespread seed-associated fungal genus [36] often implicated in competitive interactions with *Fusarium* [42]. Although *Cladosporium* was one of the most abundant epiphytic fungal genera isolated from the seed surface of both weed species in our study, it was much less frequently encountered among endophytic mycobiota samples. In fact, only seeds collected at a single site in 2020 for *A. fatua* and in 2021 for *E. crus-galli* contained *Cladosporium* in the endosphere, whereas the majority of samples from the seed surface of both weed species in both years contained representatives from this genus. A similar distinction was also observed in the case of *Vishniacozyma*, abundant on the surface of *E. crus-galli* seeds but absent from the endosphere. Such distinction between seed endosphere and surface concerning *Cladosporium* and *Vishniacozyma* colonization could be caused by the lower ability of these fungi to penetrate the seed coat or by lower competitiveness in relation to endophytes. However, further studies are required to test these speculations. Both *Cladosporium* and *Vishniacozyma* have also previously been reported as potential antagonists of *Fusarium* [42].

*Epicoccum*, a fungal genus previously described for its biocidal potential against seeds of *E. crus-galli* [43], was also among the most abundant genera in our study. Contrary to *Cladosporium* and *Vishniacozyma,* which were predominantly isolated from the seed surface, *Epicoccum* was much more frequently isolated from the endosphere, suggesting that different compartments of the seed provide a specific ecological niche for these fungi.

*Sarocladium* isolates identified as *S. strictum* (previously known as *Acremonium strictum*) [44] were isolated from the seeds of both plant species in both years, while those identified as *S. bacillisporum* were isolated only from *E. crus-galli* in 2021. In this study, *Sarocladium* was mostly isolated from the seed surface, but it also occurred in the endosphere. *S. strictum* is a pathogenic species causing *Acremonium* wilt in plants. However, its role as an endophyte needs to be studied better. *S. strictum* isolates obtained from maize kernels could produce mycotoxins, as well as antimicrobial and insecticidal compounds [45].

Isolates from the genus *Aureobasidium* were identified as *A. pullulans*, but in two cases, Protax-fungi search identified the isolates as *Selenophoma* sp., a genus that can be grouped in the same clade as *Aureobasidium* [46]. *Aureobasidium* was isolated from the seeds of both plant species in both years, although in 2020, only from *A. fatua*. This species is described as a floral pathogen [47] and an antagonist to *Fusarium culmorum* [48].

The isolates from the genus *Bipolaris* were identified as *B. sorokiniana* (synonym *Cochliobolus sativus*) by NCBI search and as *Drechslera* by Protax-fungi analysis. *D. sorokiniana* is considered a synonym of *B. sorokiniana,* and the latter is known as the causal agent of black point disease, which reduces seed quality and germination [49], as well as wheat seedling blight [50]. Other species from this genus, *Drechslera avenacea* and *B. sorokiniana,* were isolated from *A. fatua* seeds [21], and both fungi were pathogenic to *A. fatua*, but only *D. avenacea* was species-specific. Since *B. sorokiniana*, other *Bipolaris* species, and *Drechslera* species were isolated from the seeds of cultivated grasses [37], it is plausible that there is no species-specificity. In this study, *B. sorokiniana/Drechslera* was isolated only from *E. crus-galli* and not from *A. fatua* in both collection years, indicating that the applied methodology was suitable for isolating this fungal species. However, its absence in our sample pool might mean that *B. sorokiniana/Drechslera* is either rare or absent from *A. fatua* in Latvia. In future studies, it will be necessary to test the pathogenicity of this isolate to *E. crus-galli*.

*Microdochium* spp. can be hosted by different cereal crops and grasses, but in this study, it was isolated only from *E. crus-galli*. *M. nivale* is a causal agent of snow mold in *Poaceae* [51]. Colonization of seeds by *Microdochium* usually does not affect germination or seedling health, and the fungus only affects weakened plants [52]. Taxonomic designation as *M. bolleyi* was assigned to our isolates by both search methods, but low genetic diversity was reported for the ITS region, so RPB2 and the β-tubulin sequences would be preferable for species identification [51]. *M. bolleyi* was reported as an endophyte in herbaceous plant *Fagonia cretica*, capable of producing compounds with antibacterial and antifungal properties [53].

*Moesziomyces* is a genus of smut fungi that infects *Poaceae* plants. We isolated it in 2021 from seeds of both plant species. NCBI search identified the isolate from *E. crus-galli* as *Pseudozyma aphidis*, a synonym for *Moesziomyces aphidis* [54], while that isolated from *A. fatua* was *Moesziomyces bullatus*. Protax-fungi search identified all isolates as *M. bullatus*. The asexual (yeast) morph of this genus (previously known as *Pseudozyma*) is non-pathogenic, can be isolated from different plants and environmental samples, and can even be antagonistic to pathogenic fungi. In contrast, the sexual morph is parasitic, and *E. crus-galli* is considered the type of host of *M. bullatus* [54]. This distinction can explain the occurrence of this fungus in the seeds of both plant species. Contrary to a few widespread and omnipresent fungal genera, most fungal isolates were present in only specific collection sites or isolated from a very limited number of samples (Table 2). Among these less-frequent fungi were several yeast genera, *Rhodotorula, Sampaiozyma, Sporobolomyces, Filobasidium,* and *Papiliotrema* that, due to their seed-colonizing ability and low pathogenicity towards crops [55], have also been suggested as possible weed biocontrol agents. The ability to produce an antifungal toxin was reported in *Bullera alba* (basionym *Sporobolomyces albus*) [56]. The presence of many species with varying properties suggests that the fungal community is assembled on the surface and in the endosphere of the seeds after attaining equilibrium between different antagonistic fungi and, potentially, other microorganisms. The interactions of these fungi should be further studied to understand the potential of shifting this equilibrium to achieve the desired effect on seed fate in soil. Identification at the species level and distinction between strains are necessary to understand what determines the presence of certain fungi on seed surfaces or in the endosphere and the implications for seed germination and persistence in soil.

The advantages of the culture-based approach are that only viable microorganisms are isolated and identified, and living cultures can be stored for future reference. However, more comprehensive taxonomic profiles of seed-borne microorganisms can be obtained by NGS-based analysis of the DNA isolated from seed samples, which will be attempted in further studies.

### 4.2. Seed-Inoculation Experiment

Seeds of *E. crus-galli* were inoculated with a spore suspension of 10 different *Fusarium* isolates obtained from both *E. crus-galli* and *A. fatua* seeds to test the potential of these isolates to infect and degrade *E. crus-galli* seeds. To the best of our knowledge, *Fusarium* has not previously been tested for *E. crus-galli* seed degradation. Successful seed colonization by some of the tested isolates of *Fusarium* and subsequent decay of colonized *E. crus-galli* seeds that were observed in our experiments suggest that this widespread fungal genus holds potential for the biocontrol of *E. crus-galli*. However, fungal growth observed on the outer structures of the seeds may not result in seed decay if the hyphae do not penetrate inner seed structures, as observed in the case of *F. graminearum* infecting barley caryopses [57]. In contrast, the *Fusarium* isolate that infects seeds of *Bromus* tectorum is able to penetrate the embryo and endosperm and degrade the seed [15]. Hyphal growth was triggered by seed exudates, proposing an interesting question: do different *Poaceae* seeds produce similar compounds that can trigger infection by *Fusarium* or other fungi?

Seeds can escape the degrading effect of fungi by rapid germination. Therefore, an important property of *F. sporotrichioides* is its ability to infect seeds at low temperatures [58]. This allows the fungus to infect seeds in soil conditions early in the season before seeds have the potential to germinate. Seeds of *E. crus-galli* do not germinate at temperatures below 13 °C [26] and, consequently, can be more susceptible to fungal attack at low temperatures. *F. sporotrichioides* isolates could be further explored as a potential seed-degrading pathogen, but the host-species specificity should also be tested.

When considering the effect of microorganisms on the soil seed bank dynamic, it is difficult to separate seed fate from seedling fate because both decay and fatal germination can deplete the seed bank. Soil-borne and seed-borne fungi can infect the seedlings. For example, certain *Fusarium* strains reduce the emergence of *E. crus-galli* [17], and *F. tricinctum* increases seedling mortality in *Stipa bungeana* and *Lespedeza davurica* [59]. The ability to infect seeds and the effect of different *Fusarium* isolates on the seeds was very variable in the current study, in line with the results of other studies [17,22]. Interestingly, one of the isolates that caused the highest reduction in the number of intact *E. crus-galli* seeds (21s72) was isolated from *A. fatua* seeds, suggesting that the seed-degrading capacity is not host-species-specific. One of the possible causes of the between-year variability is the difference in the colonization rate of the seeds and the composition of the seed-borne microorganisms that can act as antagonists to the pathogens. Although the majority of analyzed seed samples were co-inhabited by *Fusarium* and *Alternaria*, both species can also exhibit competitive interactions, likely due to antagonistic toxin production [60]. The observed competitive interaction between *Fusarium* and *Alternaria* suggests that native endophytic mycobiota is a significant factor that needs to be considered when predicting the outcome of seed infections by potentially seed-degrading fungi.

## 5. Conclusions

In this study, we characterized the composition of culturable fungi from the surface and the endosphere of seeds of two commercially important weed species. Mature seeds harbored more fungi on the surface compared to the endosphere, and the described fungal genera included both potential seed pathogens and their antagonists. The artificial seed-infection experiment showed that some of the seed-borne *Fusarium* strains have the potential to degrade seeds of *E. crus-galli*, thereby influencing seed fate in a soil seed bank.

## Figures and Tables

**Table 1:** Seed-Collection Dates and Location Details.

Plant Species	Location Name	Coordinates	Date		Crop	
			2020	2021	2020	2021
*A. fatua*	Bebri	56°44′ N 25°27′ E	05.08.	01.08.	Spring wheat	Spring wheat
*A. fatua*	Gaigalava	56°43′ N 26°07′ E	05.08.	01.08.	Spring wheat	Spring wheat
*A. fatua*	Krimunas	56°32′ N 23°27′ E	13.08.	04.08.	Peas/spring barley	Winter wheat
*A. fatua*	Ziras	57°10′ N 21°35′ E	12.08.	n/a	Spring barley	n/a
*E. crus-galli*	Iecava	56°59′ N 24°25′ E	05.09.	23.08.	Zucchini	Maize
*E. crus-galli*	Salgale	56°37′ N 23°58′ E	01.09.	25.08.	Spring oats	Potato
*E. crus-galli*	Stelpe	56°52′ N 24°55′ E	05.09.	23.08.	Spring wheat	Spring wheat

**Table 2:** Fungi isolated from *A. fatua* and *E. crus-galli* seeds and identified at the genus and species level using NCBI Blast search and Protax-fungi algorithm. No species is given where NCBI search resulted in multiple species with the same match for the query sequence. Frequency of occurrence was calculated as the summary incidence in seed samples from each species population in each year divided by the total number of seed samples, seven in *A. fatua* and six in *E. crus-galli*. A value of 1 means that the genus/species was isolated from each species population in both years.

Frequency of Occurrence				Fungal Genera/Species	
*A. fatua*		*E. crus-galli*			
Endosphere	Seed Surface	Endosphere	Seed Surface	Protax-Fungi	NCBI
0.00	0.14	0.00	0.17	*Acremonium*	*Acremonium sclerotigenum*
0.43	0.43	0.83	0.83	*Alternaria*	*Alternaria*
0.00	0.43	0.00	0.33	*Aureobasidium pullulans*	*Aureobasidium melanogenum*
0.00	0.00	0.00	0.50	*Bullera alba*	*Bullera alba*
0.14	0.71	0.17	0.83	*Cladosporium*	*Cladosporium*
0.00	0.14	0.00	0.00	*Claviceps*	*Claviceps*
0.00	0.00	0.33	0.00	*Drechslera*	*Bipolaris sorokiniana*
0.00	0.00	0.17	0.00	*Davidiella*	*Davidiella*
0.29	0.00	0.00	0.00	*Didymella*	*Didymella pomorum*
0.00	0.14	0.00	0.00	*Dioszegia*	*Dioszegia hungarica*
0.14	0.00	0.83	0.17	*Epicoccum*	*Epicoccum nigrum*
0.00	0.00	0.00	0.33	*Filobasidium magnum/oeirense*	*Filobasidium magnum/oeirense*
0.71	0.43	0.50	0.83	*Fusarium*	*Fusarium*
0.00	0.00	0.00	0.17	*Holtermannia*	*Holtermanniella*
0.00	0.00	0.00	0.50	*Microdochium bolleyi*	*Microdochium bolleyi*
0.0	0.14	0.17	0.17	*Moeszyomyces bullatus*	*Moeszyomyces bullatus/Pseudozyma aphidis*
0.00	0.00	0.00	0.17	*Mycosphaerella*	*Mycosphaerella*
0.00	0.00	0.00	0.33	*Papiliotrema*	*Papiliotrema baii*
0.00	0.00	0.00	0.33	*Penicillium*	*Penicillium*
0.00	0.00	0.00	0.17	*Peniophora*	*Peniophora*
0.00	0.14	0.00	0.00	*Phaeoisariopsis*	*Cladosporium*
0.00	0.00	0.17	0.00	*Phaeosphaeria*	*Parastagonospora*
0.00	0.00	0.00	0.17	*Pseudocercospora*	*Cladosporium*
0.14	0.43	0.00	0.33	*Rhodotorula*	*Rhodotorula glutinis/mucilaginosa/babjevae*
0.00	0.00	0.00	0.17	Sampaiozyma ingeniosa *	*Rhodotorula ingeniosa*
0.14	0.57	0.33	0.50	*Sarocladium strictum*	*Sarocladium strictum*
0.00	0.14	0.00	0.00	*Selenophoma*	*Aureobasidium pullulans/subglaciale*
0.00	0.43	0.00	0.00	*Sporisorium*	*Sporisorium graminicola*
0.00	0.71	0.00	0.17	*Sporobolomyces*	*Sporobolomyces roseus/ruberrimus*
0.00	0.57	0.00	0.67	*Vishniacozyma tephrensis*/unknown/*carnescens*	*Vishniacozyma tephrensis/victoriae/carnescens*

* Until 2015—*Rhodotorula ingeniosa*.

**Table 3:** Fusarium isolates used for inoculating seeds of *E. crus-galli* in laboratory conditions.

Isolates	Year of Isolation	Plant Species	Plant Population	Habitat	Species
20s02	2020	*A. fatua*	Bebri	Epiphyte	*F. avenaceum*
20s11	2020	*A. fatua*	Krimunas	Epiphyte	*F. sporotrichioides*
20s16	2020	*E. crus-galli*	Iecava	Epiphyte	*Fusarium* sp.
20s23	2020	*E. crus-galli*	Salgale	Epiphyte	*Fusarium* sp.
21s19	2021	*E. crus-galli*	Stelpe	Endophyte	*F. culmorum*
21s23	2021	*E. crus-galli*	Iecava	Endophyte	*F. avenaceum*
21s28	2021	*E. crus-galli*	Stelpe	Endophyte	*F. avenaceum*
21s41	2021	*E. crus-galli*	Stelpe	Epiphyte	*F. avenaceum*
21s61	2021	*A. fatua*	Gaigalava	Endophyte	*F. sporotrichioides*
21s72	2021	*A. fatua*	Bebri	Epiphyte	*F. sporotrichioides*

## Data Availability

Data sharing not applicable.
